# Integration of mRNA and miRNA Analysis Sheds New Light on the Muscle Response to Heat Stress in Spotted Sea Bass (*Lateolabrax maculatus*)

**DOI:** 10.3390/ijms252212098

**Published:** 2024-11-11

**Authors:** Cong Liu, Haishen Wen, Yuan Zheng, Chong Zhang, Yonghang Zhang, Lingyu Wang, Donglei Sun, Kaiqiang Zhang, Xin Qi, Yun Li

**Affiliations:** Key Laboratory of Mariculture, Ministry of Education (KLMME), Ocean University of China, Qingdao 266003, China

**Keywords:** *Lateolabrax maculates*, heat stress, expression regulation, mRNA-miRNA regulatory network

## Abstract

Temperature is a crucial environmental factor for fish. Elevated temperatures trigger various physiological and molecular responses designed to maintain internal environmental homeostasis and ensure the proper functioning of the organism. In this study, we measured biochemical parameters and performed mRNA–miRNA integrated transcriptomic analysis to characterize changes in gene expression profiles in the muscle tissue of spotted sea bass (*Lateolabrax maculatus*) under heat stress. The measurement of biochemical parameters revealed that the activities of nine biochemical enzymes (ALP, γ-GT, AST, GLU, CK, ALT, TG, LDH and TC) were significantly affected to varying degrees by elevated temperatures. A total of 1940 overlapping differentially expressed genes (DEGs) were identified among the five comparisons in the muscle tissue after heat stress. Protein–protein interaction (PPI) analysis of DEGs indicated that heat shock protein genes (*HSPs*) were deeply involved in the response to heat stress. In addition, we detected 462 differential alternative splicing (DAS) events and 618 DAS genes, which are closely associated with sarcomere assembly in muscle, highlighting the role of alternative splicing in thermal response regulation. Moreover, 32 differentially expressed miRNAs (DEMs) were identified in response to heat stress, and 599 DEGs were predicted as potential target genes of those DEMs, generating 846 DEG–DEM negative regulatory pairs potentially associated with thermal response. Function enrichment analysis of the target genes suggested that lipid metabolism-related pathways and genes were regulated by miRNAs. By analyzing PPIs of target genes, we identified 28 key negative regulatory pairs, including 13 miRNAs (such as lma-miR-122, lma-miR-200b-5p and novel-miR-444) and 15 target genes (such as *hspa13*, *dnaja1*, and *dnajb1a*). This study elucidates the molecular mechanisms of response to high-temperature stress and offers valuable information for the selection and breeding of heat-tolerant strains of spotted sea bass.

## 1. Introduction

Water temperature is fundamental to the life activity of fish, regulating numerous physiological and biochemical processes. Dramatic changes in temperature can profoundly impact survival, behavior, distribution, reproduction and growth [1,2,3,4]. With the onset of global warming and the resultant effects of human activities, fish are increasingly susceptible to damage from heat stress. When faced with changes in environmental temperature, fish typically initiate multiple physiological reactions to maintain homeostasis [5,6]. Therefore, exploring the critical genes and regulators related to the thermal stress response will not only help decipher the molecular mechanisms underlying fish responses to heat stress, but also provide potential molecular elements for improving heat tolerance and artificially breeding heat-tolerant strains [7].

Advancements in next-generation sequencing have enabled the investigation of the molecular mechanisms underlying heat tolerance in fish. Several publications have described candidate genes or pathways related to the heat stress response. For instance, RNA-seq analysis has illustrated the molecular effects of thermal stress on Atlantic salmon (*Salmo salar*) [8], highlighting the important role of molecular chaperones and the endoplasmic reticulum (ER) in responding to heat stress. Additionally, the *UBIQ*, *CDKN1B*, and *HSP70* genes were up-regulated under different degrees of high temperature in *Gillichthys mirabilis* [9]. Studies on gene expression changes in other fish species under high temperatures have been conducted, including pikeperch (*Sander lucioperca*) [10], grass carp (*Ctenopharyngodon idellus*) [11,12], and two Antarctic fishes (*Notothenia rossii* and *Notothenia coriiceps*) [13]. Interestingly, genes involved in alternative splicing (AS) can also be detected in the transcriptomic data. As a widespread epigenetic mechanism in eukaryotes, AS facilitates mRNA and protein diversity [14] and is rigorously modulated, with patterns that vary under different stressors [15]. It has been shown that the AS patterns of some functional genes with a core role in the cold stress response in three-spine stickleback (*Gasterosteus aculeatus*) were altered [16]. In Nile tilapia (*Oreochromis niloticus*), comparative transcriptome analysis uncovered AS regulatory functions in heart tissue under acute hypoxic stress [17]. However, AS events related to heat stress have only been documented in a few fish species [18,19], and the specific biological functions of most AS genes are yet to be fully understood.

MicroRNAs (miRNAs), approximately 22 nucleotides (nt) in length, are a class of endogenously conserved non-coding RNA molecules [20]. They function at the post-transcriptional level to regulate gene expression by binding to the 3′-UTRs of mRNAs, thereby inhibiting translation and promoting mRNA degradation to prevent the production of the target protein [21,22]. Previous studies have shown that various biological and metabolic processes are closely related to miRNAs, validating their key roles in coping with various stressors [23,24,25,26]. For instance, in genetically improved farmed tilapia (GIFT, *O. niloticus*), mRNA and miRNA expression profiles revealed that lipid and carbohydrate metabolism pathways were involved in the response to elevated temperatures [27]. Similarly, in Tibetan naked carp (*Gymnocypris przewalskii*), thermal stress was investigated by combining mRNA and miRNA sequencing analyses, revealing that some heat shock protein genes (*HSPs*) were part of the regulation network, demonstrating the regulation of heat shock proteins by DEMs [28]. In rainbow trout (*Oncorhynchus mykiss*), miRNA profiling studies have identified several miRNAs, including miR-301a and Let-7, which are engaged in thermal adaptation [29,30]. Similar results have been found in other aquatic organisms, including Senegalese sole (*Solea senegalensis*) [31], sea urchin (*Strongylocentrotus intermedius*) [32], and *Marsupenaeus japonicus* [33]. Generally, miRNAs are non-negligible regulators involved in adaptation to high-temperature environments.

As a dominant commercial fish species, spotted sea bass (*Lateolabrax maculatus*) is widely cultivated and generates enormous market values in China. However, it exhibits poor tolerance to dramatically higher water temperatures in summer, leading to substantial economic losses [34]. Previous studies have typically focused on organs such as the liver, gill, kidney, and intestine [35,36,37], overlooking the potentially crucial roles of muscle tissue. Muscle tissue, accounting for 50 percent of body weight in most fish species, is considered a central component of the heat stress response [38]. Although several reports in the literature demonstrate positive responses of muscle tissue to thermal shock in other fish species [39,40,41], a systematic investigation of the molecular mechanisms of the reaction of the spotted sea bass to heat stress has not yet been undertaken. In this study, RNA-seq and miRNA-seq were performed to elucidate the molecular mechanisms in the muscle tissue of spotted sea bass in response to elevated temperature. The findings of this study will enhance our understanding of the molecular mechanisms of fish responses to high-temperature stress and provide useful insights for breeding new heat-tolerant strains.

## 2. Results

### 2.1. Effects of Heat Stress on Biochemical Parameters

Biochemical parameters are commonly used as indicators to assess the physiological status of fish. As shown in Table 1, ALP, GLU and γ-GT activities tended to decrease with increasing water temperature, while ALT, AST, CK and LDH activities tended to increase. Specifically, ALP activity was significantly decreased from the control to H72, with a minimum at H24. Similarly, γ-GT activity showed a significant decrease, remaining low and stable from H6 to H72. The GLU content showed a gradient decrease with the duration of heat stress. ALT activity did not change before H48, then increased significantly. Both AST, LDH and CK activities increased after thermal stress, with AST and LDH activities increasing initially and then decreasing, while CK activity showed a sustained increase. TC and TG showed a trend of increasing and then decreasing. In general, biochemical parameters were affected by high-temperature stress to varying degrees.

### 2.2. Overview of RNA-Seq Data

In this research, we conducted RNA-seq analysis on spotted sea bass exposed to heat stress. As shown by the statistical results, 810,554,018 raw reads were detected from 18 transcriptome libraries, and 767,304,696 clean reads passed the quality control assessment. Q20 (the value of Phred was more than 20) and Q30 (the value of Phred was more than 30) of sequencing reads were higher than 95.57% and 89.72%, respectively, and the G/C content averaged 50.23%. Alignment with the reference genome showed that the overall mapping ratio for all samples ranged from 88.44% to 96.61% (Supplementary Table S3).

### 2.3. Identification of DEGs

**Figure 1:**
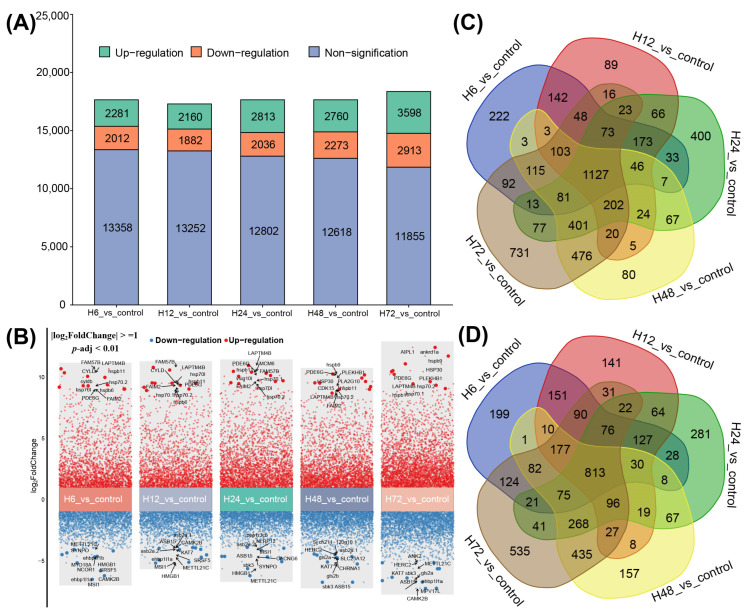
Differentially expressed genes (DEGs) at different time points after heat stress. (**A**) Histogram of statistics of the number of up-regulated, down-regulated and non-significant-difference genes in each comparison group. (**B**) Volcano plot of top 10 genes with DEGs in each comparison group. (**C**) Venn diagram of up-regulated DEGs. (**D**) Venn diagram of down-regulated DEGs.

As illustrated in Figure 1, 4293, 4042, 4829, 5033 and 6511 DEGs were identified in the five comparisons, respectively. Specifically, 2281, 2160, 2813, 2760 and 3589 DEGs were significantly up-regulated, while 2012, 1882, 2036, 2273 and 2913 DEGs were down-regulated (Figure 1A). Volcano plots of the five comparison groups are displayed in Figure 1B, with the top 10 DEGs labeled, indicating that some up-regulated (*hspb11*, *hsp70.2*, *PDE6G* and *LAPTM4B*) and down-regulated (*METTL21C* and *sbk3*) genes were remarkably differentially expressed in most comparison groups. Furthermore, 1940 common genes were identified, including 1127 up-regulated (Figure 1C) and 813 down-regulated (Figure 1D) genes, which were involved in the response to heat shock at all sample points.

### 2.4. PPI Network Construction and the Expression Profiles of Hub Genes

**Figure 2:**
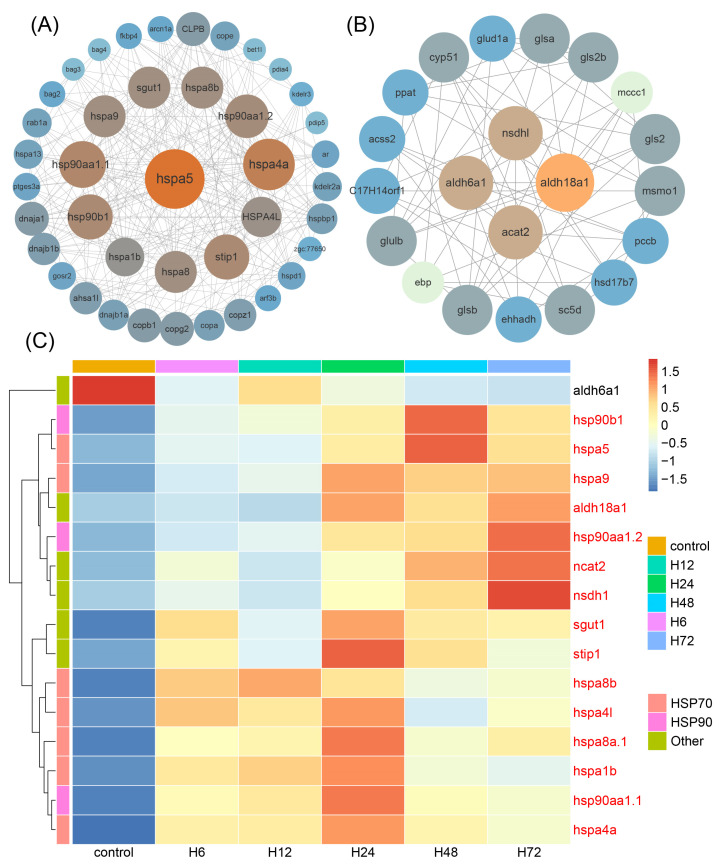
(**A**) Cluster 1 and (**B**) Cluster 2 display the PPI network of common DEGs. The color of the nodes represent the connectivity degree. (**C**) Expression profiles of hub genes of spotted sea bass after heat stress. Genes with up-regulated expression are marked in red.

To explore the pivotal genes involved in the response to high temperature in spotted sea bass, PPI networks were constructed using the 1940 commonly identified DEGs. The whole network was analyzed by MCODE, and two notable clusters with high connectivity degrees were selected for display (Figure 2). We identified 41 nodes (279 edges) in Cluster 1 (Figure 2A) and 21 nodes (64 edges) in Cluster 2 (Figure 2B). The majority of the highly linked genes in Cluster 1 were *HSPs*, including *hspa5*, *hspa4a*, *hsp90aa.1.1*, *hsp90aa.1.2*, *hsp90b1*, *HSPA4L*, *hspa8*, *hspa8b*, *hspa1b*, and *hspa9*, indicating the essential role of *HSPs* in coping with thermal stresses. In Cluster 2, four highly linked genes (*aldh18a1*, *aldh6a1*, *nsdh1* and *acat2*) were also identified (Figure 2B). The expression profiles of these hub genes showed significant up-regulation, with the exception of the *aldh6a1* gene (Figure 2C).

### 2.5. DAS Identification, Functional Enrichment Analysis and Validation

**Figure 3:**
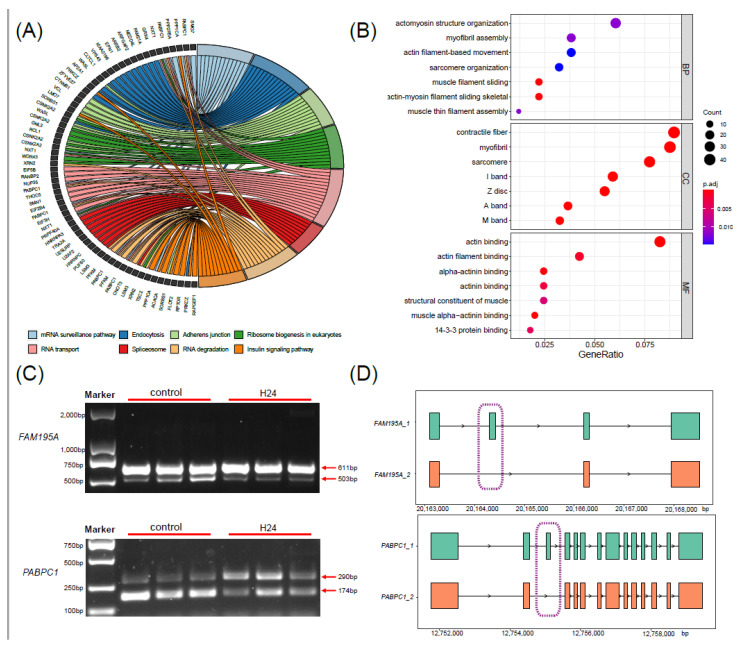
Functional enrichment and validation results for differential alternative splicing (DAS) genes in muscles after heat stress. (**A**) KEGG enrichment analysis results for DAS genes. Significant pathways are linked with their involved genes via various color ribbons. (**B**) GO enrichment analysis results for DAS genes. (**C**) Verification of DAS genes by PCR. (**D**) AS patterns of two validated genes. Purple frames represent the exon in where the AS occurs.

To identify the DAS genes in muscle, DAS events were analyzed across five comparison groups. As shown in the results, we detected 462 DAS events after heat exposure, including 84, 128, 437, 424 and 249 DASs in the five comparisons, which were generated from 73, 107, 360, 367 and 212 genes (a total of 618 genes) (Table 2). However, we only detected ES and MXE types of AS events, with ES being the most prevalent (accounting for 90.24%). To investigate the function of the 618 DAS genes, we performed KEGG and GO enrichment analyses. The KEGG functional enrichment analysis indicated that the spliceosome, the mRNA surveillance pathway, RNA transport, endocytosis, the adherens junction, RNA degradation, ribosome biogenesis in eukaryotes, and insulin signaling pathways were significantly enriched (Figure 3A). The main GO terms were associated with development, differentiation and assembly of muscle tissue, including actin binding, muscle alpha–actinin binding, the I band, the M band, myofibril assembly and skeletal muscle thin filament assembly (Figure 3B).

For validation of the RNA-seq analysis results, qPCR and RT-PCR experiments were conducted. In detail, six DEGs (*hsp70.2*, *hsp90b1*, *hspa5*, *METTL21C*, *MRTO4*, and *NOP56*) were randomly selected for qPCR at 6 h, 12 h, 24 h, 48 h and 72 h (Supplementary Figure S2). The expression patterns of the selected DEGs, as revealed by qPCR, displayed a high correlation with the RNA-seq analysis results (*R* = 0.95–0.97). In addition, we selected *FAM195A* and *PABPC1*, two predicted ES-type DAS genes, for the verification of AS events. The RT-PCR results indicated that the size of the amplification products was consistent with the AS results (Figure 3C). The alternative splicing patterns of these two genes are shown in Figure 3D.

### 2.6. Identification and Characteristics of miRNAs in the Muscle

For the identification of miRNAs potentially involved in responding to high temperatures, 79,600,949 raw reads were generated from the Illumina sequencing platform (Supplementary Table S4), with Q30 values ranging from 95.79% to 97.24%. Following processing and filtering, we obtained a total of 74,430,390 clean reads, and the statistical information of these clean reads is summarized in Supplementary Table S5. The length distribution showed that 22 nt was the most abundant, followed by 21 and 23 nt (Supplementary Figure S3A). These findings are consistent with the common length features of miRNAs [4]. After aligning to the spotted sea bass genome, the comparison rates for clean reads ranged from 88.66% to 94.52% (Supplementary Table S5). For the annotation of small RNAs, all clean reads were compared with various recorded sequences, including rRNA, tRNA, snRNA, snoRNA, other ncRNA, repeat and mRNA sequences. A total of 28,004,779 valid reads remained for the identification of known and novel miRNAs (Supplementary Table S5). As a result, 938 miRNAs (including 816 conserved and 122 novel miRNAs) were discovered. Among these, 617, 632, 663, 654, 622, and 631 miRNAs were identified in six small RNA libraries, including 596, 606, 631, 625, 591, and 608 known miRNAs, as well as 21, 26, 32, 29, 31, and 23 novel miRNAs, respectively (Supplementary Figure S3B).

### 2.7. Differential Expression Analysis of miRNAs

**Figure 4:**
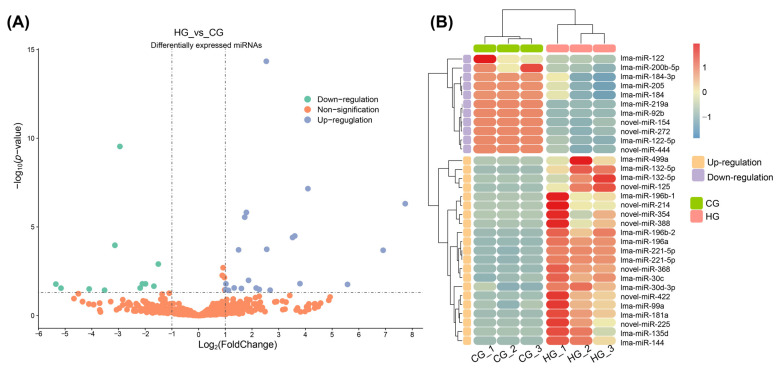
Differential expression analysis of miRNAs under heat stress. (**A**) The volcano plot of differential expression analysis between the CG and HG. (**B**) Heatmap of expression levels for DEMs of the CG and HG.

After performing differential expression analysis, we identified 32 significantly DEMs, including 21 up-regulated and 11 down-regulated miRNAs (Figure 4A). Of these, 22 miRNAs are conserved miRNAs and 10 miRNAs are novel miRNAs. Eight conserved miRNAs exhibited down-regulated expression, while fourteen conserved miRNAs showed up-regulated expression. Among the novel DEMs, three novel miRNAs showed down-regulated expression and seven novel miRNAs exhibited up-regulated expression. The relative expression levels of DEMs in each sample are shown in a heatmap (Figure 4B). To distinguish conserved and novel DEMs, novel RNAs are temporarily named as “novel” and given random numbers, and the mature sequences of the DEMs are displayed in Supplementary Table S6.

### 2.8. Target Gene Prediction of DEMs and Functional Enrichment Analysis

**Figure 5:**
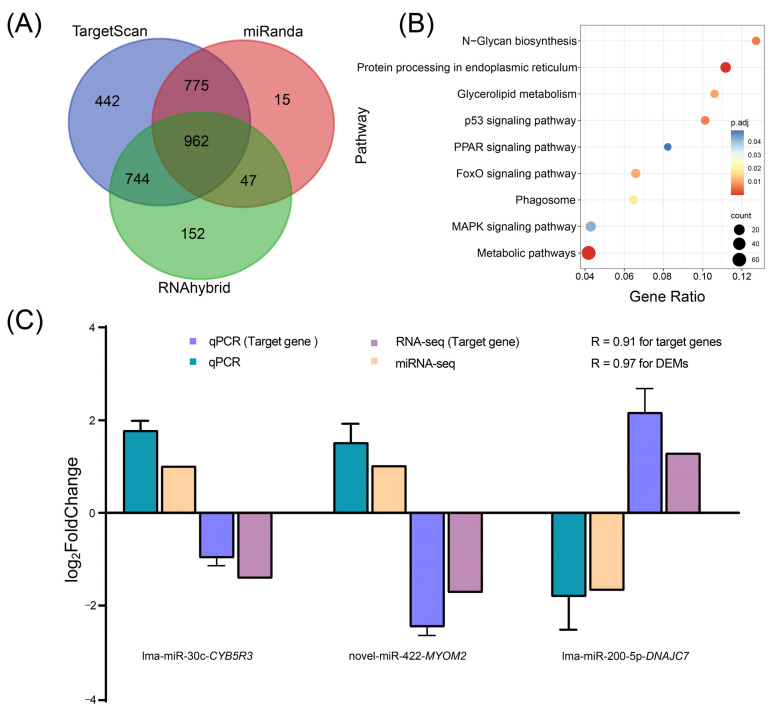
(**A**) Venn diagram of target gene prediction of DEMs. Different colors indicate different prediction tools. (**B**) KEGG pathway enrichment analysis of target genes. (**C**) Validation of DEMs and negative regulation relationships with target genes by qPCR.

Combining the mRNA expression profiles, candidate target DEGs of DEMs were predicted. As a result, 962 DEGs were predicted as target genes, with 599 DEGs ultimately selected due to negative regulation by miRNAs (Figure 5A). To further investigate the function of target genes, we performed KEGG pathway enrichment analysis. The results indicated that protein processing in the ER, the FoxO signaling pathway, the MAPK signaling pathway, metabolic pathways, the p53 signaling pathway, N-glycan biosynthesis, glycerolipid metabolism, the phagosome and the PPAR signaling pathway were significantly enriched (Figure 5B).

For validation of miRNA analysis, we randomly selected six DEMs and three DEGs (genes predicted to be targets of DEMs) for qPCR. The results suggested that changes in the expression levels exhibited similar tendencies, with expression correlation values of 0.91 and 0.97 between the qPCR and bioinformatics analyses for DEMs and target DEGs, respectively (Figure 5C and Figure S4). Moreover, three miRNAs and their target genes showed opposite expression patterns (Figure 5C), confirming the reliability of the study.

### 2.9. Regulatory Network of DEMs–DEGs

To further explore the regulatory mechanisms of DEMs, a DEM–DEG regulatory network was constructed using 32 DEMs and 599 target genes, generating 846 negative regulatory pairs (Supplementary Table S7). All pairs of regulatory relations are plotted in Supplementary Figure S5, including 310 regulatory relationship pairs mediated by 21 up-regulated miRNAs (Supplementary Figure S5A) and 536 regulatory relationship pairs regulated by 11 down-regulated miRNAs (Supplementary Figure S5B), forming a sophisticated regulatory expression network.

**Figure 6:**
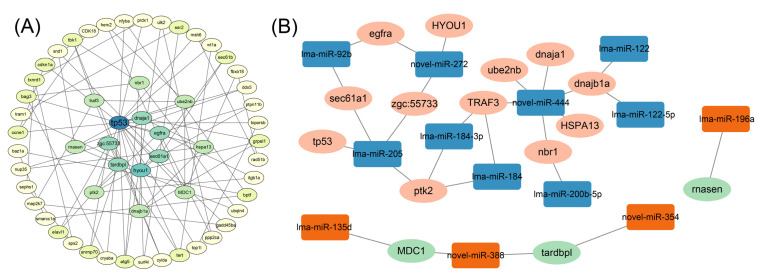
Hub target genes and regulatory network in spotted sea bass coping with thermal stress. (**A**) Identification of hub target genes. Different colors indicate the degree of connectivity. (**B**) Hub regulatory network of DEMs–DEGs. Green ellipses represent down-regulated expressed DEGs, plink ellipses represent up-regulated expressed DEGs, yellow squares represent up-regulated expressed DEMs, and blue squares represent down-regulated expressed DEMs.

To better understand the hub regulatory network, PPI analysis was conducted to identify the hub target gens. As a result, 15 target genes were identified as hub genes, including *tp53*, *dnaja1*, *egfra*, *sec61a1*, *hyou1*, *tardbpl*, *zgc:55733*, *TRAF3*, *nbr1*, *ube2nb*, *hspa13*, *MDC1*, *dnajb1a*, *ptk2*, and *rnasen* (Figure 6A). A crucial regulatory network for key target genes was then constructed, comprising 13 miRNAs and 15 target genes, generating 28 negative regulatory pairs (Figure 6B).

### 2.10. Overview of the Key DEMs, DEGs and Functional Pathways

**Figure 7:**
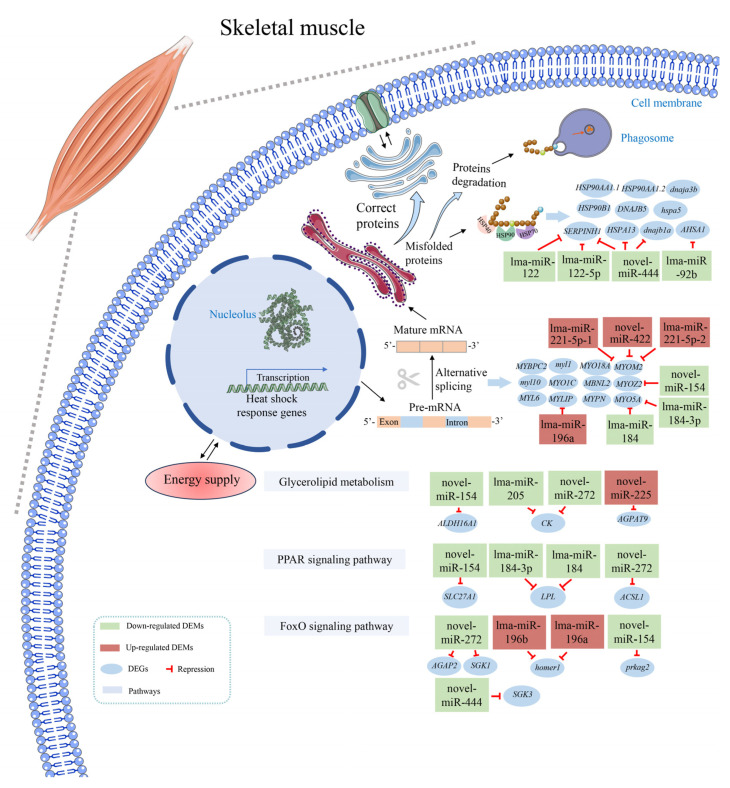
Schematic diagram of predicted molecular mechanism in the thermal response in muscle tissue of spotted sea bass.

Based on the comprehensive bioinformatics analysis of RNA-seq and miRNA-seq, combined with the predicted negative regulatory relationships of DEMs–DEGs and the functional enrichment results, a putative schematic diagram is illustrated. This diagram summarizes the pathways, DEGs and DEMs engaged in coping with thermal stress in the muscle tissues of spotted sea bass (Figure 7). In detail, we speculated that there are three predominant regulatory mechanisms: protein processing in the ER involving *HSPs*, AS events related to the sarcomere assembly of muscle, and lipid metabolism-related pathways associated with energy supply (Figure 7), which will be discussed in the Section 3.

## 3. Discussion

Dramatic changes in ambient temperature can directly or indirectly affect physiological and metabolic processes in fish [42]. Fishes exposed to high temperature can trigger a cascade of severe reactions, including heat shock, depressed immune responses, disease, developmental issues, reproductive challenges and impaired growth [43]. The spotted sea bass, a widely cultured fish in China, is particularly susceptible to heat stress during the summer months, resulting in growth and developmental impairment, as well as declines in yield and quality. However, few studies have explored the molecular mechanisms underlying heat tolerance in spotted sea bass. In this study, we conducted RNA-seq and miRNA-seq analyses of muscle tissues from spotted sea bass to provide insights into the molecular responses induced by high temperatures and elucidate multidimensional regulatory mechanisms.

Heat shock proteins (*HSPs*), a class of chaperone proteins, are essential for widespread stress resistance and environmental adaptation [44]. Their fundamental functions include mediating the formation of functional conformations of new peptide chains, contributing to intracellular protein transport and assembly, limiting the misfolding or unfolding of proteins induced by stress and assisting partially unfolded proteins to refolding into their native conformation [45]. Previous studies have revealed that *HSPs*, especially the HSP90 and HSP70 family genes, are inducible under high-temperature conditions. For instance, RNA-seq analysis showed that seven of the eight *HSP90* genes were markedly up-regulated in rainbow trout after exposure to high temperatures [46]. Similarly, five significantly up-regulated HSP70 genes were detected in large yellow croaker livers under high temperature [47]. HSP90 family genes are indispensable for the folding, assembly, trafficking and aggregation of cellular proteins, especially under heat stress conditions [44]. In our study, three *HSP90* number genes (*hsp90aa.1.1*, *hsp90aa.1.2* and *hsp90b1*) exhibited up-regulation and were significantly enriched in protein processing in the ER pathway (Figure 7), indicating that HSP90 family genes are engaged in the folding, sorting and degradation of misfolded proteins. As confirmed in catfish, *hsp90aa1*, *hsp90aa2* and *hsp90b1* genes were up-regulated, functioning in assisting protein folding and stabilization [48]. For the HSP70 number genes, it has been reported that these proteins are involved in protein folding and unfolding, offering protection to cells when exposed to high temperatures [49]. An example is the *hspa5* gene, localized to the lumen of the ER, which is a master regulator of ER homeostasis and is involved in protein folding and assembly [50]. The hspa5 protein contains nucleotide-binding and substrate-binding domains, allowing it to transfer protein into the ER, assist in ER-associated degradation, maintain protein homeostasis and protect cells from environmental stresses [50]. Moreover, *hspa5* was characterized as a potential hub gene in spotted sea bass, as evidenced by PPI analysis (Figure 2A), and the up-regulation of this gene underscores its vital role in the high temperature response. This conclusion is supported by findings in spotted seatrout (*Cynoscion nebulosus*), which showed that the *hspa5* gene in the northern population was uniquely up-regulated under acute heat stress [51]. Additionally, transcriptomic profiles of Japanese flounder (*Paralichthys olivaceus*) gills under heat stress revealed that the *hspa5* gene was among the up-regulated DEGs [52].

In addition to the HSP90 and HSP70 family genes, several other *HSPs* were significantly up-regulated after heat stress and were predicted to be target genes of differentially expressed miRNAs (DEMs), including *HSPA13*, *DNAJB1*, *DNAJA1*, *SERPINH1*, *DNAJC7*, *dnajc16* and *AHSA1* (Supplementary Table S7). Previous studies have proven that *HSPs* are targeted and regulated by miRNAs in response to elevated ambient temperatures. For instance, several *HSPs* (such as *DNAJA1* and *HSP90BB*) were predicted to be target genes of miRNAs and validated through qPCR in rainbow trout [29,30]. Additionally, numerous *HSPs* were identified as target genes of DEMs in Tibetan naked carp, suggesting that miRNAs significantly regulate the response to heat stress through *HSPs* [28]. In this study, these *HSPs* were negatively moderated by lma-miR-122-5p, novel-miR-444, lma-miR-122 and lma-miR-92b (Supplementary Table S7), implying that these miRNAs exert an instrumental regulatory function in the heat stress response.

Energy supply is critical to the biological activity of fish. From the results of the enrichment analysis of DAS genes, the insulin signaling transduction pathway was noted (Figure 3A). The main function of the insulin signaling pathway is to regulate blood glucose homeostasis in the body through glucose and lipid metabolism [53]. Besides the nutritional status of the fish, temperature significantly affects blood glucose levels [54]. Sufficient blood glucose provides an immediate energy supply to fish under high-temperature conditions. Supporting this, significant enrichment of DAS genes in the insulin signaling pathway has been documented in rainbow trout [55]. Furthermore, as an alternative source of energy supply, lipid metabolism serves as a crucial role for fish to adapt to high-temperature environments [27]. In this study, the target genes were significantly enriched in several lipid metabolism-related pathways, including the FoxO signaling pathway, glycerolipid metabolism, and the PPAR signaling pathway. The PPAR signaling pathway is pivotal in regulating lipid metabolism, encompassing adipogenesis and metabolic homeostasis [56]. In this pathway, we noted the *SLC27A1* gene targeted by novel-miR-154, which encodes a fatty acid transport protein that transports long-chain fatty acids into the cell by promoting their transport at the plasma membrane. Up-regulation of this gene may contribute to the mobilization of fat [57]. The *LPL* and *ACSL1* genes are downstream response genes of the PPAR signaling pathway. *LPL* encodes lipoprotein lipase, a pivotal enzyme in fatty acid formation, indirectly influencing lipid metabolism levels [58]. In our regulatory network, the expression of the *LPL* gene was up-regulated and targeted by lma-miR-184-3p and lma-miR-184 (Supplementary Figure S5B and Supplementary Table S7). Consequently, the *LPL* gene may increase the content of fatty acids in spotted sea bass, thereby promoting lipid metabolism under heat stress. Similar trends have been observed in tilapia and Tibetan naked carp under acute heat stress, where the *LPL* gene targeted by some significantly down-regulated miRNAs was significantly up-regulated [27,28]. Furthermore, the *ACSL1* gene encodes acyl-CoA synthetase, which functions in the conversion of free long-chain fatty acids into fatty acyl-CoA esters. It participates in the heat stress response by regulating fatty acid metabolism, as proven in juvenile turbot [59], Jinhu grouper (*Epinephelus fuscoguttatus* ♀ × *E. tukula* ♂) [60] and *Trachinotus ovatus* [61]. In this study, the expression level of the *ACSL1* gene was observed to be up-regulated and negatively regulated by novel-miR-272, suggesting that miRNAs are involved in fatty acid degradation by regulating the *ACSL1* gene. Collectively, ensuring a sufficient energy supply by regulating insulin and lipid metabolism-related signaling pathways is a vital molecular mechanism in response to heat stress in spotted sea bass.

Alternative splicing (AS) acts as a regulator of genes at the post-transcriptional level by generating mRNA and protein variants that vary in structure and function, playing a vital role in cellular and physiological adaptations [62]. Given its widespread involvement in fish responses to various harsh external environments, including cold [16], hypoxia [17], disease [63], and salinity [64], the AS mechanism is crucial for coping with heat stress. In our study, AS changes induced by heat stress were analyzed through transcriptome analysis of muscle tissue in spotted sea bass. We discovered exon skipping (ES) events to be the most frequent among the five typical AS types. Similar results have been found in other fish species, including common carp [65], spiny chromis (*Acanthochromis polyacanthus*) [66], turbot, tongue sole (*Cynoglossus semilaevis*), and rainbow trout [67]. Moreover, several studies on spotted sea bass have demonstrated the potential role of AS in responding to various ambient stresses, including hypoxia, alkalinity and salinity in the gill or liver [34,37,64]. Our results highlighted the significant regulatory role of AS in response to high-temperature stress. Notably, the KEGG functional enrichment analysis of genes with AS showed significant enrichment of the spliceosome pathway (Figure 3A). Spliceosomes, nuclear organelles responsible for deleting non-coding introns from messenger RNA precursors (pre-mRNAs) and reassembling exons to generate protein isoforms [68], exhibit alterations in several important components under heat stress conditions, potentially influencing pre-mRNA splicing. Similar findings were reported in catfish [63] and rainbow trout after thermal stress [19], where genes undergoing DAS were significantly enriched in the spliceosome. Interestingly, GO enrichment analysis exhibited a remarkable correlation with the sarcomere assembly of muscle (Figure 3B). These GO terms, including the A band, I band, M band, Z-disk, and skeletal muscle thin filament assembly, were proven to be functional in muscle sarcomere assembly during proliferation and differentiation stages in a previous study [69]. Furthermore, we also identified numerous genes related to skeletal muscle development from the DAS gene and DEG–DEM regulatory network (Figure 7), including *MYBPC2*, *myl1*, *MBNL2*, *MYOM2*, *myl10*, *MYO1C*, *MYO18A*, *MYOZ2*, *MYL6*, *MYLIP*, *MYPN*, and *MYO5A*. In particular, the *MYOM2* (Myomesin 2) gene, a major component of the vertebrate myofibrillar M band [70], was regulated by three miRNAs, simultaneously. It has also been found that *MYOM2* plays an important role in skeletal muscle cell development and differentiation in black rockfish (*Sebastes schlegelii*) [71] and European eel (*Anguilla anguilla*) [72]. Given this, it is reasonable to infer that AS is involved in the regulation of sarcomere assembly in the muscle of spotted sea bass in response to heat stress.

## 4. Materials and Methods

### 4.1. Ethics Statement

For the welfare of animals, all experiments were approved by the respective Animal Research and Ethics Committees of Ocean University of China (Permit Number: 20141201), and fishes were dissected under advanced anesthesia using MS-222 (200 mg/L). The fish used for the experiment were commercially available, and no endangered or protected species were included in this research.

### 4.2. Experimental Design and Fish Sampling

To investigate the influence of elevated temperature on spotted sea bass, wild fish (body length: 22.60 ± 1.14 cm, body weight: 102.19 ± 16.65 g) were acquired from the Weihai coastal region (Weihai, China). The acute heat stress challenge experiment was performed after a month of domestication in a square pool with a volume of approximately 25 m^3^ under a, seawater temperature of 15 ± 1 °C, pH value of 8 ± 0.5, dissolved oxygen content of more than 7 mg/L and salinity of 24 ± 0.1, at Shuangying Aquatic Seeding Co., Ltd. (Lijin, China). Fish were not fed for 24 h before exposure to high temperature, then 60 individuals were randomly assigned to three replicates of homemade net cages for the high-temperature challenge, with a density of 20 individuals in each replicate. A heating tube at the bottom of the container was used as a temperature moderator. Prior to the formal experiments, a preliminary trial was conducted in which 20 fish were placed in a same net cage and the temperature was gradually increased to 31 °C from 15 °C at a rate of 1 °C per hour. It was observed that mortality exceeded 50% within less than 12 h. A 15 °C temperature shift may be considered the critical threshold for this group of samples. Consequently, the ultimate stress temperature was determined to be 30 °C. At the start of the experiment, the seawater temperature increased at an average rate of 1 °C per hour from 15 °C to 30 °C. After about 15 h, the temperature was maintained at 30 ± 0.5 °C to monitor changes in fish reactions. During the heat challenge, all other ambient conditions were as described above for acclimatization and the tested fish were starved. After anesthetization with an MS-222 bath, the blood and muscle tissue of three individuals from each replicate were sampled at acclimatization (before heating, 15 °C), 6 h (timing from 30 °C), 12 h, 24 h, 48 h and 72 h, these being named the control, H6, H12, H24, H48 and H72 groups, respectively (Supplementary Figure S1). Blood samples were extracted immediately from the tail vein of the spotted sea bass. After standing for 24 h at 4 °C, serum was collected from blood samples by centrifugation at 5000 r/min for 10 min. The collected muscle tissue and serum were frozen in liquid nitrogen and stored under −80 °C. No fish mortality occurred during the experimental process.

### 4.3. Biochemical Parameters Assays

To detect the effect of high temperature on biochemical parameters, nine parameters were measured, including alkaline phosphatase (ALP), γ-glutamyl transferase (γ-GT), aspartate aminotransferase (AST), glucose (GLU), creatine Kinase (CK), alanine aminotransferase (ALT), triglyceride (TG), lactate dehydrogenase (LDH), and total cholesterol (TC). Equal volumes of serum from three individuals in each replicate were pooled into one sample to reduce individual differences. Enzyme activity was calculated using commercially available kits, following the manufacturer’s protocols of the Mindray BS-180 full-automatic biochemical analyzer (Mindray, Shenzhen, China). Significance testing between samples was conducted using one-way analysis of variance (ANOVA).

### 4.4. RNA Isolation, Library Preparation and Sequencing

TRIzol reagents (Invitrogen, Carlsbad, CA, USA) were used to extract total RNA from muscle tissue. Subsequently, NanoDrop 2000 and Fragment Analyzer 5400 were used to assess the concentration and integrity of the total RNA, respectively. To minimize the adverse impact of individual differences on the results, equal amounts of total RNA from three individuals in each replicate were pooled into one sample.

For transcriptome sequencing, we constructed 18 sequencing libraries (three replicates at six sampling time points) using the NEBNext Ultra^TM^ RNA Library Prep Kit (NEB, Ipswich, MA, USA). The library preparations were used to generate 150 bp paired-end reads. For miRNA sequencing, the libraries were prepared based on the extracted total RNA using NEBNext^®^ Multiplex Small RNA Library Prep Set (NEB, USA). Similarly to RNA-seq, the library preparations were employed to generate 50 bp single-end reads on the Illumina Novaseq 6000 platform. However, to reduce costs, we only sequenced the control and heat-treated (heat treatment for 24 h) groups, named CG and HG, respectively.

### 4.5. Identification of DEGs and PPI Network Construction

Fastp v0.23.2 software [73] was used to remove adapter sequences and low-quality raw sequencing reads, generating high-quality clean reads. The clean reads were then aligned to the reference genome sequence of spotted sea bass (PRJNA407434) using Hisat2 v2.2.0 software [74]. The raw count matrices were calculated using the featureCounts program [75]. To normalize gene expression levels, the count matrices were converted to fragments per kilobase of transcript per million fragments mapped (FPKM) using StringTie v2.2.1 software [76]. Differential expression analysis was carried out using DEseq2 v1.30.1 (R package) [77] to identify DEGs among the five comparisons (H6 vs. control, H12 vs. control, H24 vs. control, H48 vs. control and H72 vs. control). The significant threshold was set to |log2FoldChange| ≥ 1 and an adjusted *p*-value ≤ 0.01.

A protein–protein interaction (PPI) network for shared DEGs across all comparison groups was constructed to identify the hub genes using String (https://cn.string-db.org/ (accessed on 1 November 2024)). We considered a significant interaction with the setting of a high confidence score > 0.7. Cytoscape (http://www.cytoscape.org/ (accessed on 1 November 2024)) was used to predict the PPI network, and the connectivity degree was counted by Cyto-Hubba v0.1 to identify the potential hub gene. MCODE v2.0.0, a Cytoscape plug-in, was operated to search for significant modules with default parameters.

### 4.6. Identification of DAS Events

Differential alternative splicing (DAS) events were identified using rMATS v4.1.2 software [78], a specialized computational tool for analyzing alternative splicing (AS) events. The likelihood ratio test was performed to calculate significance at the inclusion levels. The significant threshold for DAS events was set to a false discovery rate (FDR) < 0.05. Subsequently, genes involved in the DAS events were extracted to illustrate their potential function.

### 4.7. miRNA Identification and Differential Expression Analysis

Raw reads were filtered using Cutadapt v3.4 [79] and FASTX-Toolkit v0.0.14 software to remove reads with adapters, reads containing more than 5 N bases, low-quality reads (Q ≤ 20), reads shorter than 18 nt, reads longer than 35 nt and polyA reads. The clean reads were then mapped to the spotted sea bass reference genome using Bowtie v1.3.1 software [80] with the following main parameters: -v 1 -k 1. The mapped reads were annotated using the Rfam database [81], cDNA and spotted sea bass repeat sequences. The identification of miRNAs was carried out using the remaining reads. Reads that perfectly matched mature miRNAs and hairpin sequences in miRbase v22.1 [82] were considered known conserved miRNAs. Novel miRNAs were predicted using miRDeep2 v0.1.3 software [83] with the following criteria: a score ≥ 1.0, an estimated signal-to-noise > 15 and a significant randfold *p*-value < 0.05. The expression levels of miRNA were normalized based on reads per million mapped reads (RPM). Differentially expressed miRNAs (DEMs) were determined using DESeq2, with the significant threshold set to an adjusted *p*-value ≤ 0.05 and |log2FoldChange| ≥ 1.

### 4.8. Interaction Analysis of mRNA and miRNA

Three tools were employed to predict the target genes for DEMs: miRanda v3.3a with the main parameters -sc 140 --en -10 -strict, TargetScan v5.0 with a threshold score of ≥50, and RNAhybrid [84] with default parameters. To minimize false positives, overlapping genes from the three algorithms were regarded as plausible potential target genes. Given that most well-accepted mRNAs and miRNAs interactions are negatively regulatory, negatively associated mRNA–miRNA pairs were screened for constructing interaction networks.

### 4.9. Functional Enrichment Analysis

GO and KEGG enrichment analyses were performed to investigate the biological functions of DAS events and target genes of DEMs. The enrichment module of KOBAS-i (KOBAS intelligent version) [85] was used for functional enrichment analysis, with the statistical method being the hypergeometric test and the method for FDR correction being the Benjamani–Hochberg method. An FDR < 0.05 was considered significant.

### 4.10. Validation Results by qPCR and RT-PCR

To verify the RNA-seq analysis results, the relative expression levels of several DEGs were detected using quantitative real-time PCR (qPCR). Briefly, cDNA templates were obtained from the total RNA of muscle tissue after the heat stress experiment using SPARKscript 1st Strand cDNA Synthesis Kit (SparkJade, Qingdao, China). Gene-specific primers were designed using the NCBI Primer-BLAST tool and are listed in Supplementary Table S1. A 10 μL qPCR reaction system, consisting of 3.6 μL of nuclease-free water, a 1 μL cDNA template, 5 μL SPARKscript II SYBR Green qRT-PCR Kit Mix (SparkJade, China), and 0.2 μL of each forward/reverse primer, was prepared. The reactions were run in triplicate on the StepOne Plus Real-Time PCR system (Applied Biosystems, Foster, CA, USA). The reference gene was *18s* rRNA, and the 2^−ΔΔCt^ method was used to calculate relative expression level.

For the validation of DAS events, cDNA was used as the template for RT-PCR experiment. As previously mentioned, primers were designed using the NCBI Primer-BLAST tool (Supplementary Table S1). RT-PCR experiments were conducted following the manufacturer’s instructions on a thermal cycler (Bio-Red, Pleasanton, CA, USA). PCR products were assayed by electrophoresis using a 0.5% agarose gel stained with Goldview (Yeasen Biotechnology, Shanghai, China).

To validate the miRNA results, six DEMs and three target genes were randomly selected for qPCR. For miRNA sequencing, cDNA templates were obtained from total RNA using the miRNA 1st Strand cDNA Synthesis Kit (by tailing A) (Vazyme, Nanjing, China). miRNA-specific forward primers were designed using miRNA Design V1.01 software (Vazyme, China), and the results are listed in Supplementary Table S2. A 10 μL qPCR reaction system, consisting of 5 uL of miRNA Universal SYBR qPCR Master Mix (2×), 2.6 μL of nuclease-free water, 2 μL template cDNA, and 0.2 μL of each primer, was prepared. The reference gene for miRNA was *U6*. The validation of target genes was conducted as described for the DEG validation.

## 5. Conclusions

Overall, the activities of nine biochemical parameters were significantly affected by heat stress. Additionally, high-throughput sequencing of mRNAs and miRNAs in the muscle of spotted sea bass revealed significant changes in mRNA and miRNA expression after thermal stress. Through differential expression analysis, 1940 overlapping differentially expressed genes (DEGs) were identified among the five comparisons, and 32 differentially expressed miRNAs (DEMs) were detected after heat stress. Based on target gene predictions, a DEG–DEM regulatory network was constructed. Our results showed that *HSPs*, lipid metabolism-related pathways, and AS mechanisms were crucial for thermal response. Our findings shed valuable light on the molecular regulatory mechanisms in response to thermal shock in spotted sea bass, contributing to the advancement of molecular breeding programs for heat-tolerant strains and offering useful information to improve the management of fish affected by high-temperature stress.

## Figures and Tables

**Table 1:** Effects of heat stress on nine biochemical parameters of serum.

Parameters	Control	H6	H12	H24	H48	H72
ALP	263 ± 7.37 ^a^	12.4 ± 2.21^cd^	18.9 ± 0.45 ^bc^	9.24 ± 3.72 ^d^	14.8 ± 4.06 ^bcd^	20.4 ± 5.15 ^b^
ALT	7.04 ± 1.93 ^b^	8.89 ± 1.58 ^b^	8.16 ± 2.91 ^b^	13.5 ± 4.51 ^b^	13.6 ± 2.43 ^b^	21.1 ± 6.52 ^a^
AST	21.2 ± 4.80 ^c^	164 ± 29.56 ^b^	297 ± 5.60 ^a^	210 ± 92.5 ^ab^	125 ± 40.2 ^b^	199 ± 69.3 ^b^
CK	590 ± 91.8 ^d^	3759 ± 1289 ^bc^	3621 ± 1541 ^bc^	3058 ± 177 ^c^	4880 ± 164 ^b^	11,710 ± 820 ^a^
GLU	33.1 ± 1.16 ^a^	20.0 ± 3.66 ^b^	13.7 ± 2.34 ^c^	9.83 ± 1.57 ^d^	3.10 ± 0.40 ^e^	1.86 ± 0.45 ^e^
γ-GT	16.4 ± 1.39 ^a^	0.30 ± 0.10 ^b^	0.53 ± 0.23 ^b^	0.60 ± 0.35 ^b^	0.73 ± 0.12 ^b^	0.53 ± 0.31 ^b^
LDH	123 ± 15.6 ^c^	513 ± 2.36 ^a^	602 ± 94.4 ^a^	354 ± 69.6 ^b^	305 ± 92.3 ^b^	392 ± 82.9 ^b^
TC	4.28 ± 0.96 ^b^	6.61 ± 1.04 ^a^	5.45 ± 0.78 ^ab^	7.04 ± 0.57 ^a^	7.23 ± 1.51 ^a^	5.72 ± 1.62 ^ab^
TG	1.08 ± 0.55 ^b^	1.23 ± 0.21 ^b^	1.35 ± 0.06 ^ab^	1.84 ± 0.31 ^a^	1.50 ± 0.23 ^ab^	1.09 ± 0.28 ^b^

Note: a, b, c, d, and e represent the difference between sample points (*p* < 0.05).

**Table 2:** Statistics of differential alternative splicing (DAS) events detected in muscle after heat stress.

AS Types	H6 vs. Control	H12 vs. Control	H24 vs. Control	H48 vs. Control	H72 vs. Control
Exon skipping (ES)	60 (11:49)	113 (24:89)	414 (93:321)	395 (95:300)	211 (40:171)
Mutually exclusive exon (MXE)	24 (5:19)	15 (5:10)	23 (16:7)	29 (13:16)	38 (14:24)
Total DAS events	84	128	437	424	249
DAS gene	73	107	360	367	212

## Data Availability

Publicly available datasets were analyzed in this study. The sequencing data were been deposited in the NCBI database, accession numbers PRJNA1071322 and PRJNA1071344.

## Supplementary Materials

The following supporting information can be downloaded at https://www.mdpi.com/article/10.3390/ijms252212098/s1.
