# Supplementary material for: Integration of mRNA and miRNA Analysis Sheds New Light on the Muscle Response to Heat Stress in Spotted Sea Bass (Lateolabrax maculatus)
Source: Int J Mol Sci. 2024 Nov 11;25(22):12098. doi: 10.3390/ijms252212098 (PMC11594061; doi:10.3390/ijms252212098)
Supplement: Supplementary file 1 [file ijms-25-12098-s001.zip › Supplementary Figure.pdf]

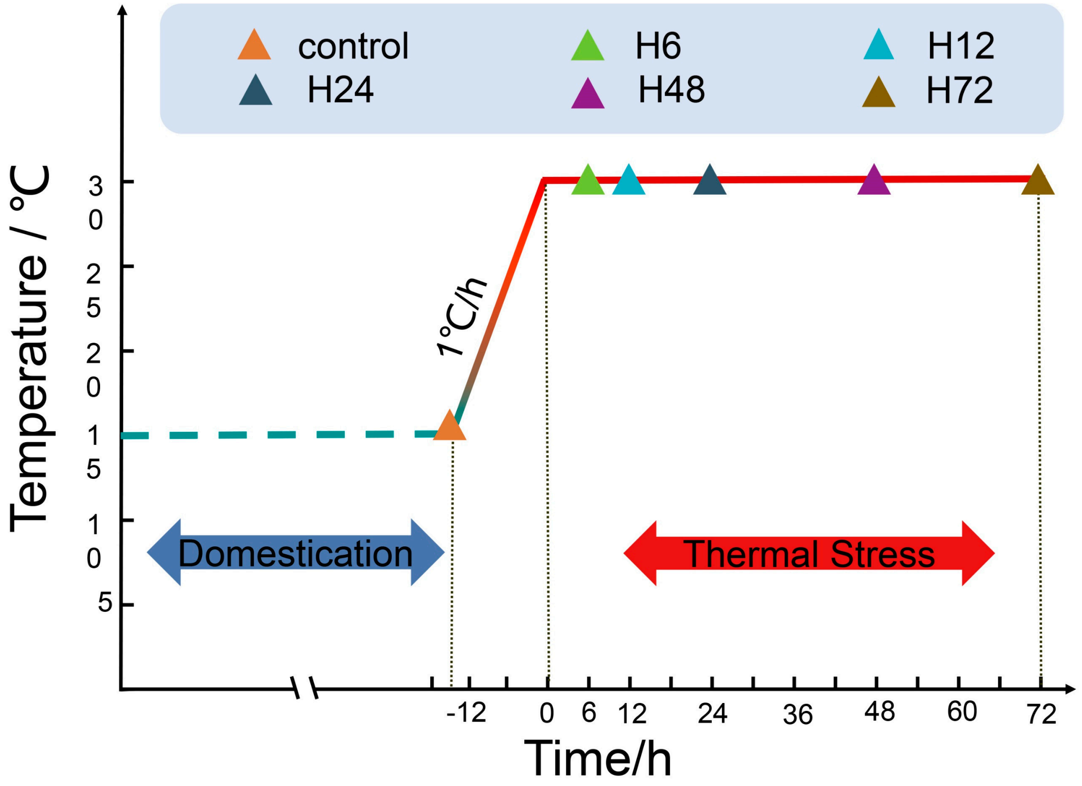

Supplementary Figure S1 Schematic diagram of sampling points for thermal stress. Different colors of triangles represent sampling points, respectively.

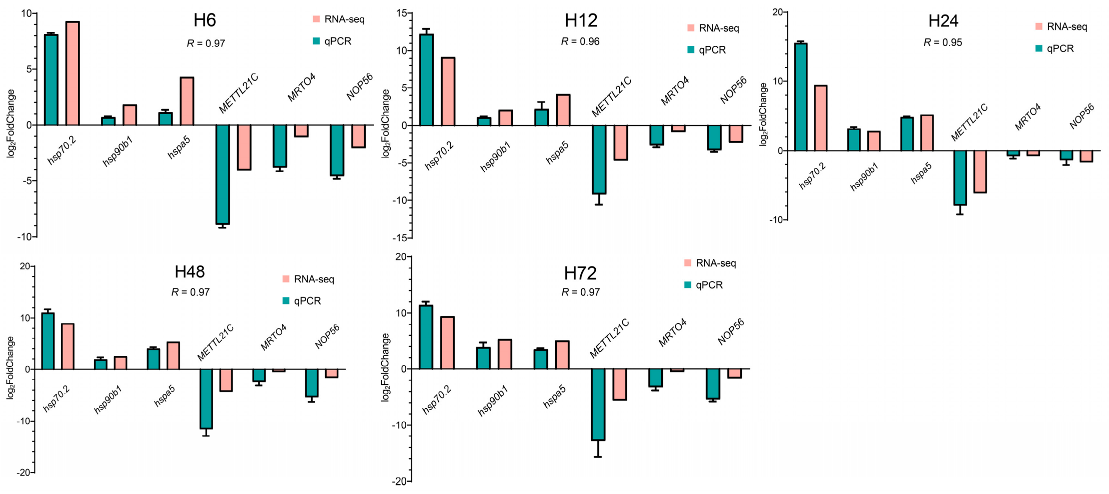

Supplementary Figure S2 Verification of RNA-seq data by qPCR.

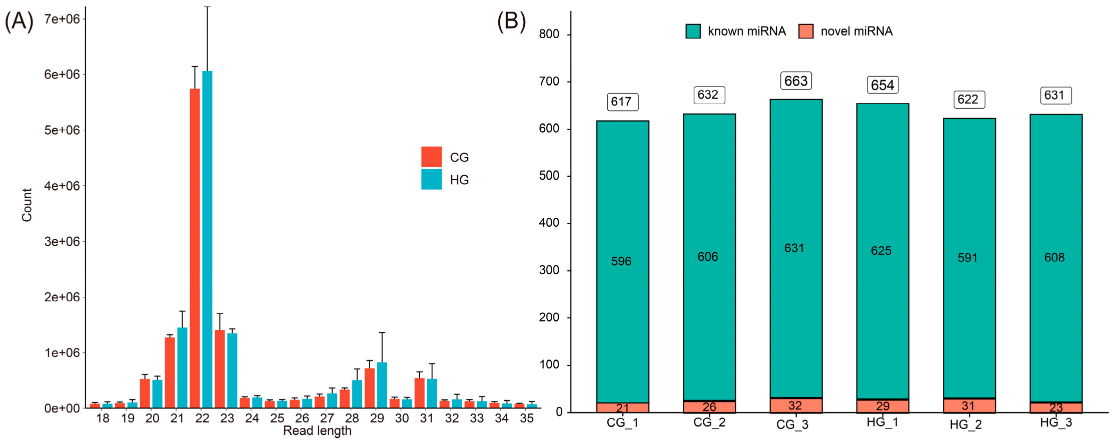

Supplementary Figure S3 Characterization of miRNAs in spotted sea bass. (A) Length distribution of the clean reads for CG and HG. (B) The number of miRNAs in CG and HG.

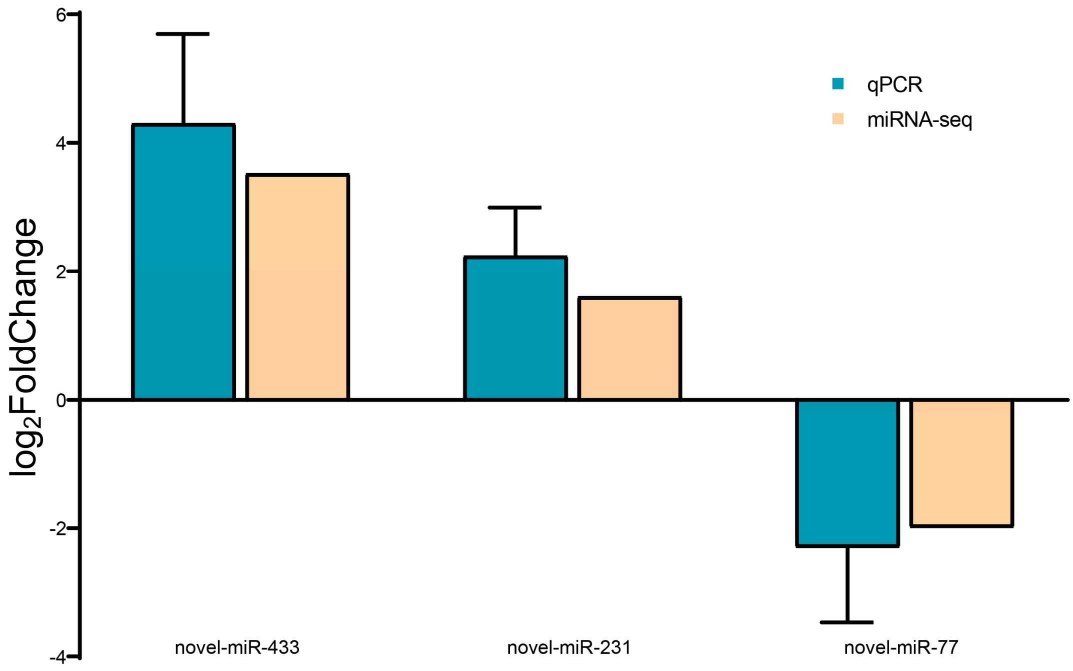

Supplementary Figure S4 Verification of three DEMs by qPCR.

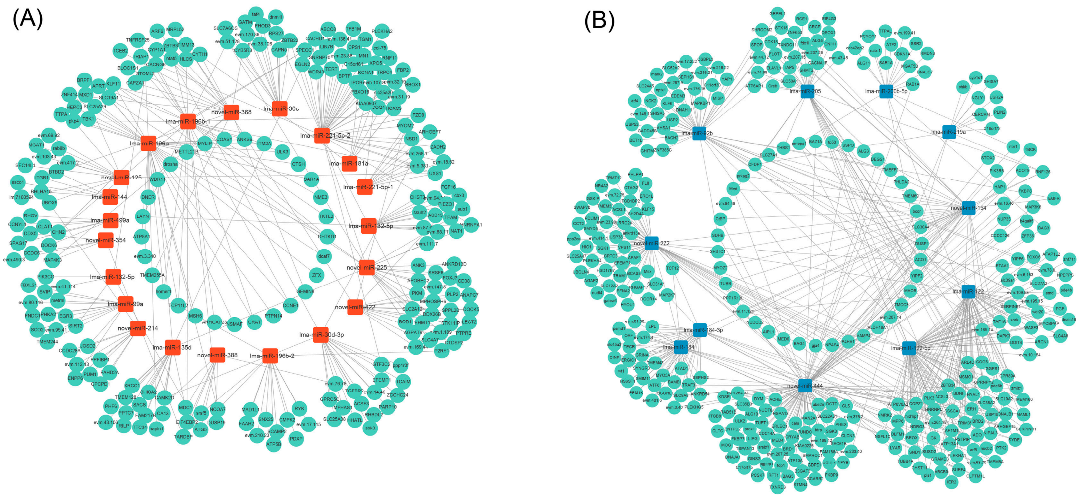

Supplementary Figure S5 Regulatory pairs of DEMs-DEGs. (A) Regulatory expression networks constructed by up-regulated DEMs and down-regulated DEGs. (B) Regulatory expression networks constructed by down-regulated DEMs and up-regulated DEGs.
